# Isolation and Characterization of Biosurfactant-Producing Bacteria from Amapaense Amazon Soils

**DOI:** 10.1155/2021/9959550

**Published:** 2021-08-16

**Authors:** Elisa Maria de Oliveira, Victor Hugo Gomes Sales, Marcelo Silva Andrade, Jerri Édson Zilli, Wardsson Lustrino Borges, Tiago Marcolino de Souza

**Affiliations:** ^1^State University of Amapá, Av. Presidente Vargas, 650 Bairro Central, 68900-070 Macapá-AP, Brazil; ^2^Federal Institute of Amapá, Campus Macapá, BR 220 km 03 Bairro Brasil Novo, 68909-398 Macapá-AP, Brazil; ^3^National Center for Agrobiology Research (Embrapa Agrobiologia), Rodovia BR-465 km 7, 23897-970 Seropédica-RJ, Brazil; ^4^Embrapa Amapá, Rodovia Juscelino Kubitschek, 68903-419 Macapá-AP, Brazil; ^5^Embrapa Tropical Agroindustry, Rua Dra. Sara Mesquita 2270, 60511-110 Fortaleza-CE, Brazil

## Abstract

The objective of this research was to perform screening of biosurfactant-producing bacteria from Amapaense Amazon soils. Floodplain- and upland-forest soils of three municipalities of the Amapá state were isolated and identified. The isolates were cultured in nutrient broth with olive oil, and their extracts were evaluated according to drop collapse, oil dispersion, emulsification, and surface tension tests. From three hundred and eighteen isolates, the 43 bacteria were selected and identified by 16S rDNA gene sequencing, indicating the presence of three different genera, *Serratia*, *Paenibacillus*, and *Citrobacter*. The extracellular biosurfactant production pointed out the 15 most efficient bacteria that presented high emulsification capacity (*E*_24_ > 48%) and stability (less than 10% of drop after 72 h) and great potential to reduce the surface tension (varying from 49.40 to 34.50 mN·m^−1^). Cluster analysis classified genetically related isolates in different groups, which can be connected to differences in the amount or the sort of biosurfactants. Isolates from *Serratia* genus presented better emulsification capacity and produced a more significant surface tension drop, indicating a promising potential for biotechnological applications.

## 1. Introduction

Microbial surfactants or biosurfactants are intracellular or extracellular metabolites of fungi and bacteria [1] classified into different structural and functional groups: lipopeptides, glycolipids, polysaccharide-protein complexes, phospholipids, neutral lipids, and fatty acids [2]. These molecules can perform different natural roles in the growth and reproduction of microorganisms [3].

Matsuyama and Nakagawa [4] observed that *Serratia marcescens* formed a giant colony when inoculated at agar medium (at 30°C for one week). In contrast, a single round colony was verified for mutant bacteria defective in wetting-agent production. They evidenced that serrawettins played a critical role in *S. marcescens* colony growth on solid-air interfaces. For *Bacillus subtilis*, the surfactin production and flagellar biosynthesis were found to be essential in swarming motility [5]. Luo et al. [6] also reported that surfactin and bacillomycin L. played important roles in the antagonistic activity and swarming motility of *B. subtilis* 916 against *R. solani* through biofilm formation and colonization. The rhamnolipids produced by *Pseudomonas aeruginosa* exhibited antimicrobial activity against several bacterial and fungal species [7]. They cause necrotic death of polymorphonuclear leukocytes, enhance cell virulence, and reduce phagocytosis susceptibility [8]. For *Pseudomonas aeruginosa*, rhamnolipids could be considered a multifunctional component of a mechanism controlling fundamental elements of microbial life [9].

The Amazon region shows rich biodiversity, comprising various elements like animals, plants, and microorganism species. Despite the intensive efforts to study this biome and the greater knowledge about its characteristics, microbial diversity remains unexplored in the Amapaense Amazon. Microorganisms play unique and vital functions in ecosystems and biosphere maintenance; therefore, Amapaense Amazon could provide microorganisms required for developing substances of biotechnological interest [10, 11]. Thus, the aim of the present research was the isolation and screening of biosurfactant-producing bacteria from the Amapaense Amazon, Brazil. In order to achieve this aim, the objectives include (1) selection of upland- and floodplain-forest soils of three municipalities using some qualitative and quantitative methods; (2) chemical and physical characterization of isolated soil samples; (3) isolation of biosurfactant-producing bacterial strains; (4) screening for biosurfactant production; and, finally, (5) identification of screened biosurfactant-producing strains by 16S rDNA gene sequencing.

## 2. Material and Methods

### 2.1. Collection Sites

**Figure 1:**
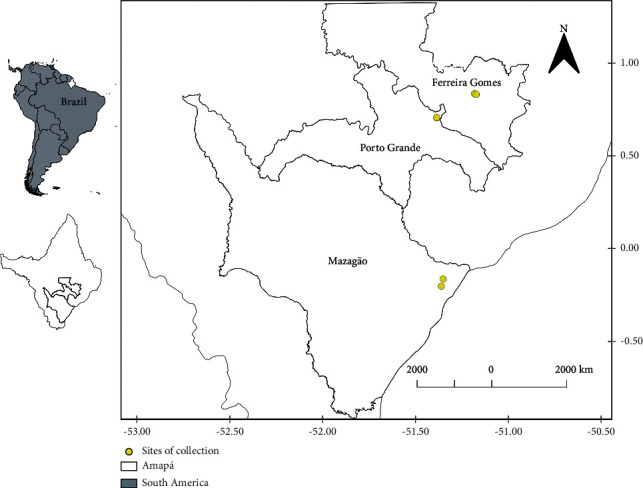
Sites of soil collection in the upland- (U-) and floodplain-forest (F) ecosystems at three municipalities: (i) Ferreira Gomes (FGU and FGF), (ii) Porto Grande (PGU and PGF), and (iii) Mazagão (MZU and MZF). Black-colored circles indicate collecting sites.

Amazonian soil samples of two different ecosystems, upland-forest (U) and floodplain-forest (F), both under equatorial forest formation, were collected in three municipalities of the Amapá State, Brazil [Ferreira Gomes (FG), Porto Grande (PG), and Mazagão (MZ)] (Figure 1). The geographic coordinates of the collection sites were as follows: FGU (N 00°50′07,7″; W 051°11′05,2″), FGF (N 00°49′49,6″; W 051°10′29,6″), PGU (N 00°42′16,5″; W 051°23′15,2″), PGF (N 00°42′24,1″; W 051°23′18,3″), MZU (S 00°09′39,0″; W 051°21′14,5″), and MZF (S 00°11′57,8″; W 051°21′47,6″). For each collection site, the ground surface was cleaned to remove plants and decomposing organic material. The soil was collected in three distinct points (in a circle with a diameter of 500 cm and depth of 20 cm), homogenized to obtain a sample of approximately 500 g, and transported to the laboratory under aseptic and refrigerated conditions.

### 2.2. Chemical and Physical Characterization of Soil Samples

The chemical analyses of the soil samples were performed in the Laboratory of Soil and Plant Physiology at Embrapa-Amapá, Brazil, according to procedures proposed by the Brazilian Agricultural Research Corporation (Table 1) [12].

### 2.3. Isolation of Biosurfactant-Producing Bacteria

To promote bacterial growth, 10 g of each homogenized soil sample was suspended in 90 mL of a peptone saline solution [0.85% NaCl; 0.1% peptone; (w·v^−1^)]. The suspension was incubated at 30°C for one hour using an orbital shaker (150 rpm) and then allowed to stand for 30 min [13]. Subsequently, supernatant aliquots of 1 mL were added to 99 mL of the nutrient broth (KASVI K25-610037, Brazil) (1.0 g of meat extract, 2.0 g of yeast extract, 5.0 g of bacteriological peptone, 5.0 g of sodium chloride, and 4.0 mL of nystatin antifungal agent per liter of distilled water; pH = 6.8 ± 0.2) and incubated with orbital shaking (150 rpm) at 30°C for 72 hours.

The incubated suspensions were serially diluted from 10^−1^ up to 10^−8^, according to the methodology described by Ozkan and Adiguzel [14]. Then, an aliquot of 100 *μ*L of each dilution was inoculated in agar nutrient (1.0 g of meat extract, 2.0 g of yeast extract, 5.0 g of bacteriological peptone, 5.0 g of sodium chloride, 15.0 g of bacterial agar, and 4.0 mL of nystatin antifungal agent per liter of distilled water; pH = 6.8 ± 0.2) and PIA (*Pseudomonas Isolation Agar*®) media (20.0 g of bacteriological peptone, 1.4 g of magnesium chloride, 10.0 g of potassium sulfate, 0.025 g of Irgasan (Ciba-Geigy), and 13.6 g of agar per liter of distilled water; pH 7,0 ± 0,20), aiming to evaluate the occurrence of *Pseudomonas* genus [15]. The plates were incubated in a BOD (QUIMIS Q 316M4, Brazil) chamber for 48 hours at 30°C. Bacterial colonies were counted in triplicate for the 10^−6^, 10^−7^, and 10^−8^ dilutions, and the results were expressed in terms of colony-forming units (CFU mL^−1^).

Bacterial phenotypic traits (colony morphological characteristics) like size, color, shape, border type, and colony relief were considered during the isolation process. Isolates were preserved under freezing (−12°C) in a solution (v·v^−1^) of 50% of nutrient broth and 50% sterilized glycerol at 20%.

### 2.4. Biosurfactant Production

Isolates were cultured in nutrient broth with 1% (v·v^−1^) olive oil for 72 hours at 30°C. After incubation, the extracts were evaluated by drop collapse, oil dispersion, and emulsification tests to identify biosurfactant-producing isolates. Isolates were registered under the code A49223C on the National Genetic Heritage Management System (SISGEN), as recommended by the Brazilian Biodiversity Law (n° 13.123/2015), and deposited at the Johanna Döbereiner Biological Resource Center (Embrapa Agrobiologia).

### 2.5. Screening of Biosurfactant-Producing Bacteria

The following selection criteria were adopted for screening of biosurfactant-producing bacteria: (i) at least two isolates from each collection site and (ii) all isolates with *E*_24_ ≥ 50%. These criteria were chosen to investigate microorganisms from all studied ecosystems.

**Figure 2:**
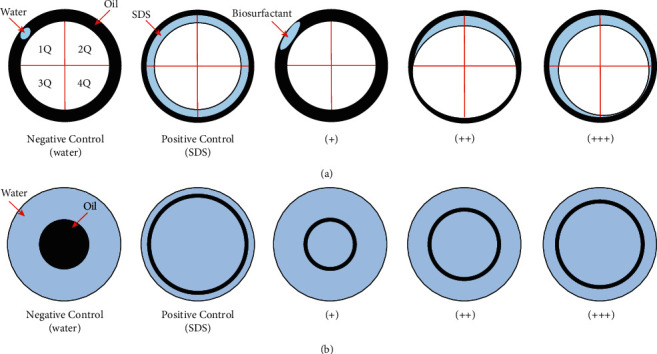
Schematic representation of the qualitative tests used to evaluate the isolated bacterial strains' capacity to produce biosurfactants: (a) drop collapse test, where 1Q, 2Q, 3Q, and 4Q represent the first, second, third, and fourth quadrants; (b) oil dispersion test.

The drop collapse and oil dispersion tests were performed using the cell culture of isolates. For drop collapse, 10 *μ*L of a burned lubricating oil was added to each well of a 96-well plate and allowed to stand for 24 hours at room temperature. Then, 10 *μ*L of the culture was added to the oil's surface, and the drop shape was observed after one minute of incubation. For the oil dispersion test, the Petri dishes were filled with distilled water (35 mL), and the burned lubricating oil (100 *μ*L) was added to the water surface. Subsequently, 10 *μ*L of the cell culture was added to the center of the oils' surface [16, 17]. In both cases, the positive control and negative control were realized with a 1% sodium dodecyl sulfate (SDS) solution and distilled water, respectively. The result was considered positive when the drop was totally or partially scattered and negative when it remained unchanged [16–18]. The activity of the produced biosurfactant was classified as weak (+), moderate (++), and strong (+++), as indicated in Figure 2.

Emulsification capacity was evaluated by adding 2.0 mL of commercial kerosene in a screw cap test tube containing 2.0 mL of cell culture, followed by vigorously mixing in a vortex (Multimixer Kasvi K40, Brazil) 3000 rpm for 2 min [19]. Measurements were performed after 24, 48, and 72 hours at room temperature. The emulsification index *E*_24h_ was calculated by the ratio between emulsion column height after 24 hours and total column height. The stability was determined considering the column emulsion height after 48 and 72 hours (*E*_48h_ and *E*_72h_), respectively.

### 2.6. Second Screening of Biosurfactant-Producing Bacteria

This screening evaluated the extracellular biosurfactant production by the selected microorganisms. Cell culture was carried out as previously described, and extracts were centrifuged at 6000 rpm for 10 min at 4°C to obtain the cell-free supernatant [20]. The supernatant emulsification was measured after 24, 48, and 72 hours, as described before.

The following selection criteria were adopted in this screening: (i) at least one isolate from each of the selected ecosystems and (ii) all isolates that show emulsification index *E*_24_ ≥ 48% and emulsification stability (did not show more than 10% of drop after 48 and 72 hours). These criteria were established to select microorganisms with potential for biotechnological processes.

Considering the extracellular selection and aiming to confirm the isolates' potential to produce biosurfactants, they were cultured again. The cell-free supernatant was used to determine *E*_24_ and surface tension. Surface tension was measured using a (KRUSS EASYDYN, Germany) tensiometer, according to the methodology described by Kuyukina et al. [15]. Before each test, the DU NUOY ring was sterilized using a Bunsen burner and calibrated with distilled water (∼70.4 ± 0.1 *m*N·m^−1^), as proposed by Du Noüy [21].

Obtained data were analyzed using a one-way analysis of variance (ANOVA) and means compared by the Tukey test at 5% significance. Principal component analysis (PCA) and hierarchical cluster analysis (HCA) based on the Euclidean distance and complete linkage method were performed using the Minitab^®^ 19 software and the methodology described by Ferreira et al. [22]. Principal component analysis (PCA) based on Euclidean distance and complete linkage method was applied to evaluate similarity among analyzed strains using surface tension (*σ*) and emulsification indexes (*E*_24_, *E*_48_, and *E*_72_ with and without cells) as descriptors. After the first PCA run, essential descriptors to describe the variance were maintained, whereas correlated ones were excluded. Surface tension (*σ*) and emulsification indexes (*E*_24_ with and without cells) were the selected descriptors. Hierarchical cluster analysis (HCA) based on the Euclidean distance and the complete linkage method was also applied to group microorganisms based on the surface tension and emulsification indexes (*E*_24_ with and without cells).

The genomic DNA extraction was carried out using the Wizard® Genomic DNA kit (Promega, Madison, WI, USA), following the manufacturer's recommendations. DNA concentration was measured by spectrophotometry at 260 nm (NanoDrop, Thermo Fisher Scientific, Waltham, MA, USA), and their integrity was verified on agarose gel at 1% (w·v^−1^; 60 V; 1 h). The 16S rDNA gene was amplified with the primers 27F 5′-AGA GTT TGA TCC TGG CTC AG- 3′ and 1492R 5′-GGT TAC CTT GTT ACG ACT T-3′. The PCR was realized on a thermal cycler (Applied Biosystems™ SimpliAmp) under the following conditions: 1.5 U Taq DNA polymerase, 1x PCR buffer (10 mM of Tris-HCl pH 8 and 50 mM of KCl), 1.75 mM of MgCl_2_, 0.25 mM of each dNTP, 0.2 *μ*M of each primer, and 1 *μ*L of the DNA template, with a total volume of 50 *μ*L. Amplification was performed using initial denaturation at 94°C for 3 min, followed by 29 cycles of denaturation at 94°C for 1 min, annealing at 58°C for 1 min, extension at 72°C for 2 min and, and a final extension at 72°C for 7 min.

Sequencing reactions were carried out using a DYEnamic™ ET Dye Terminator kit (MegaBACE™) and an automatic MegaBACE 1000 sequencer (GE Healthcare Life Sciences). The obtained sequences were deposited at the NCBI GenBank, with accessions numbers MK156425-MK156460 and MT252662-MT252668. Then the obtained sequences were compared with the National Center Biotechnology Information database (http://www.ncbi.nlm.nih.gov) using the BLAST tool [23]. Subsequently, the sequences were aligned using the Cluster W program, and the phylogenetic tree was built based on the method of maximum likelihood [24] with the aid of the Mega X software [25]. Finally, the isolates' phylogeny was analyzed using the maximum likelihood method and the Tamura–Nei model, including bootstrap analysis based on 1000 replications [24] to estimate the confidence level of the tree topology.

## 3. Results

Amazonian soil samples collected from two different ecosystems have shown a significant number of CFU. The number of counted microorganisms per milliliter (CFU mL^−1^) was 3.96 × 10^9^, 2.25 × 10^9^, 1.67 × 10^9^, 1.69 × 10^9^, 2.41 × 10^9^, and 4.95 × 10^9^ for the FGU, FGF, PGU, PGF, MZU, and MZF ecosystems, respectively. A total of 318 bacteria were isolated (Table 2). Upland-forests (FGU, PGU, and MZU) showed greater bacterial populational density than floodplain ones (FGF, PGF, and MZF), 227 and 91 isolates, respectively. The FGU alone presented 138 isolates, which corresponds to 43,4% of the total isolated bacteria.

For the drop collapse test, 237, 73, and 8 isolates presented weak, moderate, and strong activities, respectively. For the oil dispersion tests, 204, 113, and 1 isolates demonstrated weak, moderate, and strong activities, respectively.  Table 3 presents the results attained for the 43 bacterial isolates chosen in the first screening.

A total of 15 isolates were selected in the second screening (Table 2). Concerning the biosurfactants' production with and without cells, emulsification results for MZU32 and MZF02 have presented significant differences and highlighted the fundamental role of the extracellular evaluations (Table 3).

**Figure 3:**
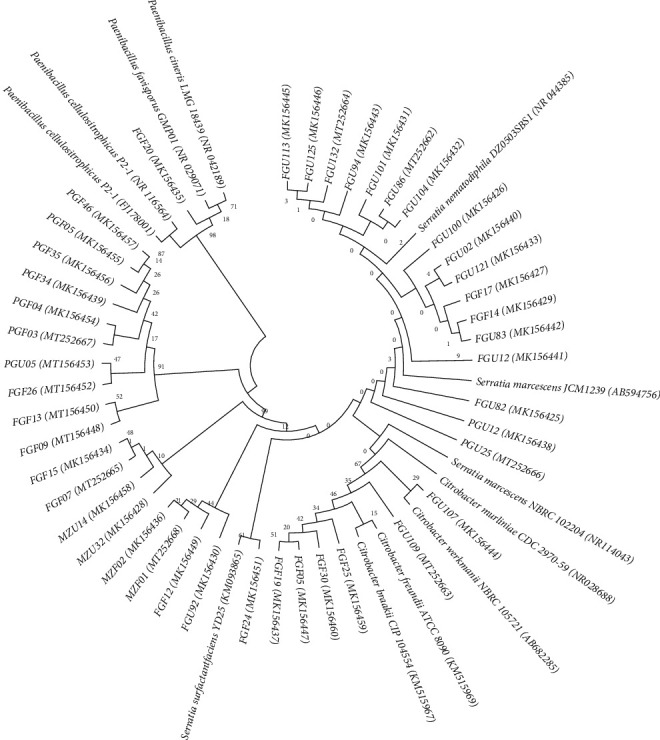
Phylogenetic analysis of the bacterial strains. The evolutionary history was inferred by using the maximum likelihood method and Tamura–Nei model [24]. The tree with the highest log likelihood (−3857.63) is shown. This analysis involved 55 nucleotide sequences. There were a total of 1439 positions in the final dataset. Evolutionary analyses were conducted in MEGA X [25].

Bacteria selected in the first screening belong to three genera: 36 from *Serratia*, 6 from *Citrobacter*, and 1 from *Paenibacillus* (Figure 3). The group comprised of PGF03, PGF04, PGF05, PGF34, PGF35, PGF46, PGU05, FGF07, FGF09, FGF12, FGF13, FGF15, FGF24, FGF26, FGU92, MZU14, MZU32, MZF01, and MZF02 presented high similarity (>99.0%) with the *Serratia surfactantfaciens* YD25 (KM093865) strain. For example, the 16S rDNA sequence identity of FGF24 (MK156451) with this strain was 99.64%. The phylogenetic analysis also showed a group of isolates (FGU12, FGU14, FGU83, FGU86, FGU94, FGU100, FGU101, FGU104, FGU113, FGU121, FGU125, and FGF17) closely related to *Serratia marcescens* JCM1239 (AB594756) strain, all of them with identity >99.0%. *Citrobacter murliniae* CDC 2970-59 (NR 028688) was the closest strain to FGU 107 (MK156444), with 99.78% similarity. The analysis placed FGU 109 (MT252663) in the vicinity of *Citrobacter braakii* CIP 104554 (KM515967), with 99.57% 16S rDNA sequence identity. FGF20 (MK156435) was placed in the neighborhood of *Paenibacillus favisporus* GMP01 (NR029071) strain with 99.49% similarity.

The 15 bacteria selected in the second screening were cultured again, and cell-free supernatant was used to determine the emulsification index (*E*_24_) and surface tension (*σ*). The variables showed significant differences (Table 4) and were compared using the Tukey test (Table 5). Eleven isolates presented *E*_24_ > 48%, whereas the surface tension ranged from 34.50 to 49.4 *m*N·m^−1^.

**Figure 4:**
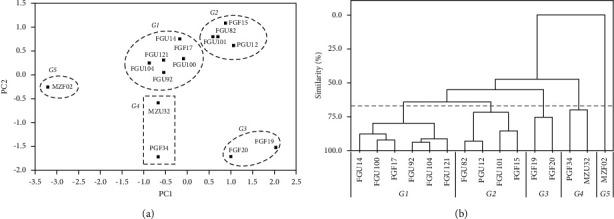
Plots of PC1-PC2 scores (a) and HCA dendrogram (b) for the biosurfactant-producing isolates based on the surface tension (*σ*) and emulsification indexes (*E*_24_ with and without cells) results. *G*1, *G*2, *G*3, *G*4, and *G*5 indicate formed groups. Fenon line is indicated by the dashed line perpendicular to the HCA similarity axis.

The score plot of the first and second components showed a separation of five distinct groups of isolates, *G*1, *G*2, *G*3, *G*4, and *G*5 (Figure 4(a)). The clustering results as a function of isolates similarity are shown in Figure 4(b). According to the established Fenon line (dashed line), five clusters were observed, reproducing the pattern observed in PCA and highlighting more details about the similarity among the isolates in each group.

## 4. Discussion

The present study was conducted to isolate and screen biosurfactant-producing bacteria from the Amapaense Amazon, Brazil. It considered upland- and floodplain-forest soils of three municipalities. In Ferreira Gomes and Porto Grande municipalities, floodplain soils presented higher pH values than upland ones. A similar result was attained by Fajardo et al. [26] who analyzed floodplain and forest soils in the Amazonas state. Only the FGF and MZU samples presented high organic matter content (>5%). The variation in soil microbial diversity and population can be affected by different factors such as pH (indicated as the best predictor of soil bacterial diversity and richness) [27], spatial influence [28], and soil organic matter [29]. Delgado-Baquerizo et al. [30] indicated that total carbon was positively related to soil bacterial diversity and was one of the most critical factors. These authors have also suggested the following: (i) bacterial diversity decreases with altitude in terrestrial ecosystems; (ii) extreme climatic conditions are the main drivers of altitude effects; and (iii) poorer bacterial diversity of southern hemisphere soils can be associated with the lower carbon content and microbial turnover rates. On the other hand, Wieder et al. [31] indicated that bacteria diversity might be limited only under very low carbon conditions. The analysis of the bacterial biodiversity is beyond the scope of this first screening of the Amapaense Amazon soil. Nonetheless, the soil samples' characterization may be helpful in future studies.

FGU101 and FGF17 isolates presented high emulsification indexes but showed strong and weak activity in qualitative tests, respectively (Table 3). This comparison illustrates that drop collapse and oil dispersion tests, as used in this work, favored the identification of biosurfactant-producing isolates but did not allow a consistent rating of the isolates' potential. On the other hand, the emulsification index (quantitative) permitted the detection of biosurfactant-producing bacteria and provided a more consistent rating of the isolated microorganisms (Table 3).

Some studies have reported the importance of applying different screening methods to prospect biosurfactant-producing microorganisms [16, 32]. In the present work, two qualitative methods (drop collapse and oil dispersion) and a quantitative one (emulsification) were applied. Further, Jain et al. [17] affirmed that the qualitative drop collapse test has a high correlation with surface tension due to the biosurfactant's ability to destabilize the liquid droplets on the oily surface. Meanwhile, Ariech and Guechi [16] also reported several isolated microorganisms that presented high, moderate, and weak capacity to collapse the oily surface. Regarding the qualitative oil dispersion test, the diameter of the central clear zone generated by the oil displacement indicates biosurfactant effectiveness. Furthermore, it has a linear relationship with the amount of biosurfactant produced [19, 32]. Various researchers have suggested that qualitative methods are more reliable for prospecting biosurfactant-producing microorganisms [16, 18].

Emulsification test is a straightforward quantitative method to prospect biosurfactant-producing microorganisms [16]. An emulsification index (*E*_24_) higher than 50% has already been used as a criterion to select biosurfactant-producing isolates. Concerning the hydrophobic compounds in various screening processes, kerosene is the most widely used one [20, 33].

Emulsification capacity is strongly affected by the produced metabolite, which also depends on the bacteria genus and producer strain [34]. Bacteria selected in this work belong to *Serratia*, *Citrobacter*, and *Paenibacillus* genera, which can produce distinct metabolites. *Serratia* is a facultative anaerobic Gram-negative bacterium that belongs to the Enterobacteriaceae family and Gammaproteobacteria class [35]. Isolates belonging to this genus were able to produce lipase (*Serratia* sp. W3), prodigiosin (*Serratia marcescens* FZSF02) [36, 37], and biosurfactant [38]. Furthermore, they can promote plant growth by solubilizing phosphates [39]. *Citrobacter* genus also belongs to Enterobacteriaceae family and Gammaproteobacteria class and can be classified as a facultative anaerobic non-lactose-fermenting Gram-negative bacteria [40]. *Paenibacillus* genus belongs to Paenibacillaceae family and Bacilli class. Their colonies are smooth and translucent, with colors ranging from light brown to white, facultative anaerobic Gram-positive, or strict aerobic [41]. Some studies also report biosurfactant production by *Paenibacillus alvei* [42], *Paenibacillus* sp. [43], *Citrobacter murliniae* [44], and *Citrobacter freundii* [45].

Determination of the surface tension reduction by bacterial cultivation medium has been recommended to confirm the ability of isolates to produce biosurfactants [46, 47]. Microorganisms able to reduce the surface tension by 20 *m*N·m^−1^, when compared with distilled water, were classified by Willumsen and Karlson [48] as promising biosurfactant producers. All the 15 selected strains decreased the broth surface tension by a value superior to the minimum established. The higher and lower surface tension reduction corresponded to 35.9 *m*N·m^−1^ (for MZF02) and 21.0 *m*N·m^−1^ (for FGF19), respectively (Table 5).

The first two principal components (PC1 and PC2) explained 78.9% of the total variance among analyzed strains. In the PC1 axis, emulsification indexes (*E*_24_ with and without cells) showed load coefficients slightly higher than surface tension one, but the three selected descriptors contributed positively to the separation of groups. Concerning PC2, *σ* presented a negative and comparatively higher load and was the most crucial variable to separate the isolates in the graph vertically. PCA analysis did not show outliers, proving that data are within the acceptable region. *G*1 represents isolates that produce biosurfactants with high emulsification capacity, which can significantly reduce the cultivation medium's surface tension. Bacteria from *G*2 are very similar to those from *G*1 but with slightly lower emulsification capacity. *G*3 group represents microorganisms that promoted the lowest surface tension reduction and do not belong to the *Serratia* genus. Finally, isolates generated from *G*4 and *G*5 groups presented low emulsification capacity and produced medium and high surface tension drop, respectively. The descriptor variation in each dendrogram cluster did not show a defined pattern, possibly due to differences among isolates. A branch on the dendrogram bottom represents a single sample, whereas the branch length linking two clusters is related to their similarity [22]. The shorter the branch is, the higher the similarity is. The groups on the left side of the dendrogram (*G*1 and *G*2) are composed of bacteria that produce metabolites with high emulsification capacity significantly reducing the medium surface tension (Figure 4(b)). The MZF02 branch length indicates low similarity in the isolate's potential to produce biosurfactants compared to the others. PCA and HCA results suggested differences in the amount of biosurfactant or the surface-active compounds by each group of bacteria, indicating that further characterizations may be useful to understand the observed pattern.

## 5. Conclusions

Biosurfactants usage has been increasing in various industrial sectors due to the growing demand for eco-friendly materials. In this work, we have isolated and characterized biosurfactant-producing bacteria from floodplain and upland Amapaense Amazon soils, Brazil. The isolates' extracts were evaluated using drop collapse, oil dispersion, emulsification, and surface tension tests, and the selected microorganisms were identified by 16S rDNA gene sequencing. 43 biosurfactant-producing bacteria belonging to *Serratia*, *Paenibacillus*, and *Citrobacter* genera were identified. Among these isolates, 15 were selected due to the higher emulsification capacity and potential to reduce surface tension. Ferreira Gomes municipality concentrated most of the isolates, and Serratia strains attained significantly higher emulsification indexes and surface tension reductions for the tested conditions. Further studies are required for the identification of metabolites produced by these isolates and for the optimization of biosurfactant production.

## Figures and Tables

**Table 1:** Chemical and physical characteristics of soil samples from the different studied ecosystems.

Characteristics of the soil samples	Ecosystem codes					
	FGU	FGF	PGU	PGF	MZU	MZF
pH	4.6	5.5	4.9	5.1	4.3	4.2
Organic matter (mg·kg^−1^)	3.55 × 10^3^	1.05 × 10^5^	2.50 × 10^4^	4.55 × 10^4^	6.45 × 10^4^	4.62 × 10^4^
Phosphorus (mg·m^−3^)	3.00 × 10^3^	4.00 × 10^3^	5.00 × 10^3^	6.00 × 10^3^	2.00 × 10^3^	2.00 × 10^3^
K^+^ (mg·m^−3^)	1.96 × 10^4^	1.96 × 10^4^	2.35 × 10^4^	2.74 × 10^4^	2.35 × 10^4^	2.35 × 10^4^
Ca^2+^ + Mg^2+^ (mg·m^−3^)	9.73 × 10^4^	9.73 × 10^4^	8.51 × 10^4^	1.09 × 10^5^	6.08 × 10^4^	4.86 × 10^4^
Ca^2+^ (mg·m^−3^)	—	1.20 × 10^5^	—	—	—	—
Al (mg·m^−3^)	1.44 × 10^5^	6.30 × 10^4^	9.89 × 10^4^	1.35 × 10^5^	1.98 × 10^5^	3.24 × 10^5^
H^+^ + Al^3+^ (mg·m^−3^)	2.14 × 10^5^	1.80 × 10^5^	1.48 × 10^5^	2.24 × 10^5^	2.96 × 10^5^	4.18 × 10^5^
SB^*∗*^ (mg·m^−3^)	1.17 × 10^5^	1.17 × 10^5^	1.09 × 10^5^	1.37 × 10^5^	8.42 × 10^4^	7.21 × 10^4^
CEC^●^ (mg·m^−3^)	3.31 × 10^5^	2.97 × 10^5^	2.57 × 10^5^	3.60 × 10^5^	3.80 × 10^5^	4.90 × 10^5^
Base saturation (%)	9	10	12	9	5	4
Al^3+^ saturation (%)	64	33	58	60	79	88
Clay (%)	10.1	34.9	19.8	17.4	35.9	25.3
Coarse sand (%)	62.5	0	48.0	37.5	10.5	24.5
Fine sand (%)	16.5	0	24.0	17.0	9.5	14.0
Silt (%)	10.9	65.1	8.2	28.1	44.1	36.2
BSSC^♦^	Sandy loam	Silty clay loam	Sandy loam	Sandy loam	Clay loam	Sandy

^♦^Brazilian System of Soil Classification; ^*∗*^SB = Ca^2+^ + Mg^2+^ + K^+^; ^●^CEC = SB + H^+^ + Al^3+^.

**Table 2:** Total number of isolates and the number of biosurfactant-producing isolates after the first and second screening stages.

Items	FGU	FGF	PGU	PGF	MZU	MZF
Total number of isolates	138	30	47	59	42	2
First screening	17	13	3	6	2	2
Second screening	7	4	1	1	1	1

**Table 3:** Drop collapse, oil dispersion, and emulsification (with and without cells) results for microorganisms selected in the first screening.

Codes of the isolated microorganisms	Drop collapse	Oil dispersion	First screening			Second screening		
			Emulsification index (%) (with cells)			Emulsification index (%) (cell-free)		
			24 h	48 h	72 h	24 h	48 h	72 h
FGU02	+	+	62.2	11.4	6.7	4.4	4.4	4.4
FGU12	+	+	64.4	53.5	51.2	15.9	6.8	6.7
FGU14	+++	++	58.1	55.8	55.8	50.0	50.0	47.7
FGU82	++	++	63.6	59.1	56.8	55.6	53.3	53.3
FGU83	+	+	51.1	51.2	48.8	15.6	15.6	15.6
FGU86	+	+	51.2	45.5	45.5	47.7	45.5	45.5
FGU92	+	+	52.3	17.8	11.4	48.9	48.9	48.9
FGU94	+	+	50.0	40.9	38.6	28.9	26.7	26.7
FGU100	+	+	53.3	44.4	44.4	53.3	53.3	53.3
FGU101	+++	+++	52.3	45.5	45.5	60.9	60.9	60.9
FGU104	+	+	50.0	46.7	46.7	48.9	48.9	48.9
FGU107	+	+	65.1	50.0	51.2	45.7	32.6	32.6
FGU109	+	+	68.2	66.7	65.9	28.9	28.9	22.2
FGU113	+	+	50.0	37.8	26.7	35.6	35.6	22.2
FGU121	+	+	50.0	48.9	48.9	51.1	51.1	51.1
FGU125	+	+	52.3	50.0	50.0	33.3	33.3	31.1
FGU132	+	+	63.6	50.0	50.0	6.7	6.7	6.7
FGF05	+	+	51.1	46.7	46.7	44.4	42.2	42.2
FGF07	+	+	52.3	52.3	50.0	50.0	2.0	2.0
FGF09	+	++	55.6	51.1	51.1	37.0	32.6	32.6
FGF12	+	+	53.3	51.1	51.1	50.0	4.3	2.2
FGF13	+++	++	50.0	36.4	31.8	15.6	15.6	13.3
FGF15	++	++	58.7	56.5	50.0	60.0	60.0	60.0
FGF17	+	+	56.5	47.8	45.7	54.3	54.3	54.3
FGF19	+	+	60.9	58.7	58.7	58.7	56.5	56.5
FGF20	+	+	50.0	50.0	50.0	55.6	55.6	55.6
FGF24	+	+	56.8	56.8	54.5	46.7	46.7	44.4
FGF25	+	+	62.2	51.1	60.0	44.4	42.2	42.2
FGF26	+	+	56.5	52.2	50.0	17.8	6.7	6.7
FGF30	+	+	55.6	53.3	53.3	23.3	16.3	14.0
PGU05	++	++	58.7	52.2	52.2	28.9	28.9	22.2
PGU12	++	++	62.8	16.3	2.3	57.8	57.8	55.6
PGU25	+	+	56.5	54.3	54.3	20.0	13.3	11.1
PGF03	+	++	54.3	52.2	52.2	9.3	9.3	7.0
PGF04	+	+	60.0	57.8	55.6	6.7	6.7	6.7
PGF05	++	+	57.8	57.8	55.6	8.9	8.9	6.7
PGF34	++	++	51.2	51.2	46.5	40.9	40.9	40.9
PGF35	++	+	54.3	52.2	52.2	31.8	29.5	29.5
PGF46	+	+	52.3	52.3	52.3	13.3	13.3	13.3
MZU14	+	+	52.3	52.3	52.3	4.4	4.4	2.2
MZU32	+++	++	63.0	63.0	63.0	40.0	40.0	37.8
MZF01	+	+	23.9	23.9	19.6	0	0	0
MZF02	++	+	21.3	12.8	12.8	44.4	44.4	44.4

**Table 4:** ANOVA results for surface tension and emulsification tests.

Source	Degrees of freedom	Sum of squares	Mean square	*F*	*p* value
	Surface tension				
Microorganisms (between)	14	938.528	67.0377	3351.89	≤0.001
Error (within)	30	0.600	0.0200	—	—
Total	44	939.128	—	—	—

	Emulsification index				
Microorganisms (between)	14	1785.57	127.541	208.93	≤0.001
Error (within)	30	18.31	0.610	—	—
Total	44	1803.88	—	—	—

**Table 5:** Surface tension and emulsification index results for the bacteria in the second screening.

Codes of the isolated microorganisms	Mean ± SD^*∗*^	
	Surface tension *σ* (*m*N m^−1^)	Emulsification index *E*_24_ h (%)
FGU14	34.93^k^ ± 0.06	51.17^f^ ± 0.71
FGU82	36.40^hi^ ± 0.00	54.67^de^ ± 0.58
FGU92	37.37^e^ ± 0.06	48.27^g^ ± 0.47
FGU100	37.20^ef^ ± 0.00	52.60^ef^ ± 0.70
FGU101	37.27^ef^ ± 0.06	60.27^a^ ± 0.81
FGU104	35.87^j^ ± 0.06	47.80^g^ ± 0.59
FGU121	36.73^gh^ ± 0.06	51.43^f^ ± 1.37
FGF15	36.13^ij^ ± 0.06	59.73^ab^ ± 0.76
FGF17	36.93^fg^ ± 0.06	54.03^de^ ± 0.25
FGF 19	49.40^a^ ± 0.20	57.73^bc^ ± 1.15
FGF20	48.63^b^ ± 0.15	55.60^cd^ ± 1.01
PGU12	38.13^d^ ± 0.06	57.27^c^ ± 0.73
PGF34	44.60^c^ ± 0.46	41.03^i^ ± 0.30
MZU32	38.50^d^ ± 0.00	39.33^i^ ± 0.85
MZF02	34.50^l^ ± 0.00	43.60^h^ ± 0.15

Mean	38.84	51.64
Coefficient of variation	0.22	1.37

^*∗*^SD: standard deviation; means followed by the same case letters in the same column do not differ from each other by the Tukey test at 5% significance.

## Data Availability

The experimental data used to support the findings of this study are included within the article.
